# Heterologous contralateral repeated bout effect: eccentric knee extensor conditioning enhances neuromuscular fatigue resistance and attenuates muscle stiffness in contralateral elbow flexors in young women

**DOI:** 10.3389/fphys.2026.1823848

**Published:** 2026-10-05

**Authors:** Chung-Chan Hsieh, Szu-Kai Fu, Yu-Lin Ning, Chin-Fang Wang, Che-Ming Liu, Chia-Hsien Yu

**Affiliations:** 1 Department of Exercise and Health Sciences, College of Kinesiology, University of Taipei, Taipei City, Taiwan; 2 Department of Martial Arts, College of Kinesiology, University of Taipei, Taipei City, Taiwan; 3 Department of Facility Planning, Ministry of Sports, Taipei City, Taiwan

**Keywords:** acoustic radiation force impulse, cross-education, eccentric exercise, exercise-induced muscle damage, heterologous protection, muscle stiffness, neuromuscular control, proprioception

## Abstract

**Background:**

The repeated bout effect (RBE) protects skeletal muscles from exercise-induced muscle damage (EIMD). While homologous contralateral RBE is well-documented, the existence of heterologous RBE—specifically whether lower limb conditioning can protect upper limb muscles—remains debated.

**Purpose:**

This study investigated whether high-intensity eccentric exercise of the knee extensors attenuates eccentric exercise-induced neuromuscular fatigue and muscle stiffness and preserves neuromuscular control in the contralateral elbow flexors.

**Methods:**

Twenty-four sedentary young women were randomized into a Knee Extensor-Contralateral Elbow Flexor (KE-CEF) group and a Control Elbow Flexor (CEF) group. The KE-CEF group performed 60 maximal eccentric contractions (MaxEc) of the non-dominant knee extensors, followed 14 days later by 30 MaxEc of the dominant elbow flexors. The CEF group performed only the elbow flexor MaxEc. Outcome measures, including maximal voluntary isometric contraction (MVC), Acoustic Radiation Force Impulse (ARFI) imaging, and the Joint Release Angle (JRA) test, were assessed up to 120 h post-exercise.

**Results:**

The KE-CEF group demonstrated significantly less MVC loss immediately post-exercise compared to the CEF group (70.00 ± 19.85% vs. 57.67 ± 8.28%; *p* = 0.029). Furthermore, prior lower limb conditioning significantly attenuated peak muscle stiffness at 48 h (ARFI: 108.25 ± 4.73% vs. 125.63 ± 6.99% in CEF) and entirely preserved JRA proprioceptive stability during peak fatigue (26.58 ± 0.78° vs. 24.75 ± 1.55°; between-group *p* = 0.001).

**Conclusion:**

These findings provide novel evidence that a single bout of lower limb eccentric exercise confers a robust heterologous protective effect on upper limb muscles, highlighting the role of global systemic and neural adaptations in cross-limb rehabilitation strategies.

## 1 Introduction

Resistance exercise, particularly unaccustomed eccentric contractions, often induces exercise-induced muscle damage (EIMD). This phenomenon is characterized by structural disruption of muscle fibers, leading to symptoms such as delayed onset muscle soreness (DOMS), prolonged loss of muscle strength, increased muscle stiffness, and reduced proprioceptive acuity (Clarkson and Hubal, 2002; Hadjisavvas et al., 2024). These impairments can significantly affect daily activities and athletic performance. However, skeletal muscle possesses a remarkable adaptive mechanism known as the repeated bout effect (RBE), where a single bout of eccentric exercise confers a protective effect against muscle damage from a subsequent bout of the same exercise (Calvo-Rubio et al., 2024). While this protection is most potent in the exercised muscle group (ipsilateral RBE), recent evidence reinforces that this adaptation can be transferred to the contralateral homologous muscle group (contralateral RBE, CL-RBE), a phenomenon often linked to the “cross-education effect” (Chen et al., 2023).

The mechanisms underlying CL-RBE are thought to involve complex neural and systemic adaptations. Neural theories propose that unilateral training enhances corticospinal excitability and modifies motor unit recruitment strategies, while systemic factors, such as cellular memory and inflammatory cytokine responses, may circulate to distant muscles to create a “pre-conditioned” environment (Ben Othman et al., 2025; Calvo-Rubio et al., 2024). While homologous CL-RBE (e.g., right arm conditioning protecting the left arm) is well-documented (Chen et al., 2023), the existence of a “heterologous” RBE—specifically, whether conditioning the lower body can induce protection in the upper body—remains a subject of debate.

Current literature regarding heterologous effects is scarce and inconsistent. For instance, Ye et al. (2021) reported that eccentric exercise of the elbow flexors failed to protect the knee flexors (Ye et al., 2021). Similarly, Chen et al. (2021) found that maximal eccentric exercise of the leg did not confer protection to the elbow flexors in young men (Chen et al., 2021). Furthermore, Jeon et al. (2022) observed no significant protective effect on muscle damage markers in heterologous muscles (Jeon et al., 2022). In contrast, other studies on cross-education suggest that unilateral training can induce global training gains or “motor overflow effects” to distant muscles under specific conditions, particularly when tasks involve maximal-intensity eccentric contractions (> 80% maximal voluntary effort) of large muscle masses that generate substantial central nervous system drive and cross-hemispheric cortical excitation (Ben Othman et al., 2020; Green and Gabriel, 2018). These discrepancies may stem from differences in training intensity, volume, or the population studied. Furthermore, most prior heterologous RBE investigations have predominantly focused on males. Females display distinct neuromuscular control strategies—such as lower maximal motor unit firing rates, different muscle activation patterns, and higher proportions of Type I muscle fibers—as well as estrogen-mediated fluctuations in sarcolemmal stability and excitation-contraction coupling (Ben Othman et al., 2025; Romero-Parra et al., 2021). Thus, there is a critical need to explore these mechanisms in women to establish whether cross-limb pre-conditioning operates under female-specific physiological profiles.

This study aims to address these gaps by investigating whether a high-intensity bout of eccentric knee extensor exercise can attenuate muscle damage and fatigue in the contralateral elbow flexors (heterologous contralateral transfer) in young women. Unlike previous studies that relied primarily on strength measures, this study incorporates novel assessment tools, including Acoustic Radiation Force Impulse (ARFI) imaging to quantify muscle stiffness and Joint Release Angle (JRA) to assess proprioceptive fatigue.

We hypothesized that prior lower limb conditioning would induce systemic or neural adaptations sufficient to enhance recovery kinetics, preserve neuromuscular control, and improve neuromuscular fatigue resistance in the heterologous upper limb. Understanding this cross-limb interaction could provide a scientific basis for novel rehabilitation protocols, allowing injured upper limbs to benefit from lower limb training when direct exercise is contraindicated.

## 2 Materials and methods

### 2.1 Study design

This study employed a randomized, controlled design. Twenty-four sedentary, healthy women were recruited and randomly assigned to two groups (n = 12 per group): the Knee Extensor-Contralateral Elbow Flexor (KE-CEF) group and the Control Elbow Flexor (CEF) group. The KE-CEF group performed maximal eccentric exercise (MaxEc) of the non-dominant knee extensors, followed 14 days later by MaxEc of the dominant elbow flexors. The CEF group performed only the dominant elbow flexor MaxEc without prior lower limb exercise. Outcome measures were assessed at baseline (Pre), immediately post-exercise (D0), and at 24, 48, 72, 96, and 120 hours post-exercise (D1–D5). A schematic illustration of the study procedure is presented in **Figure 1**.

**Figure 1:**
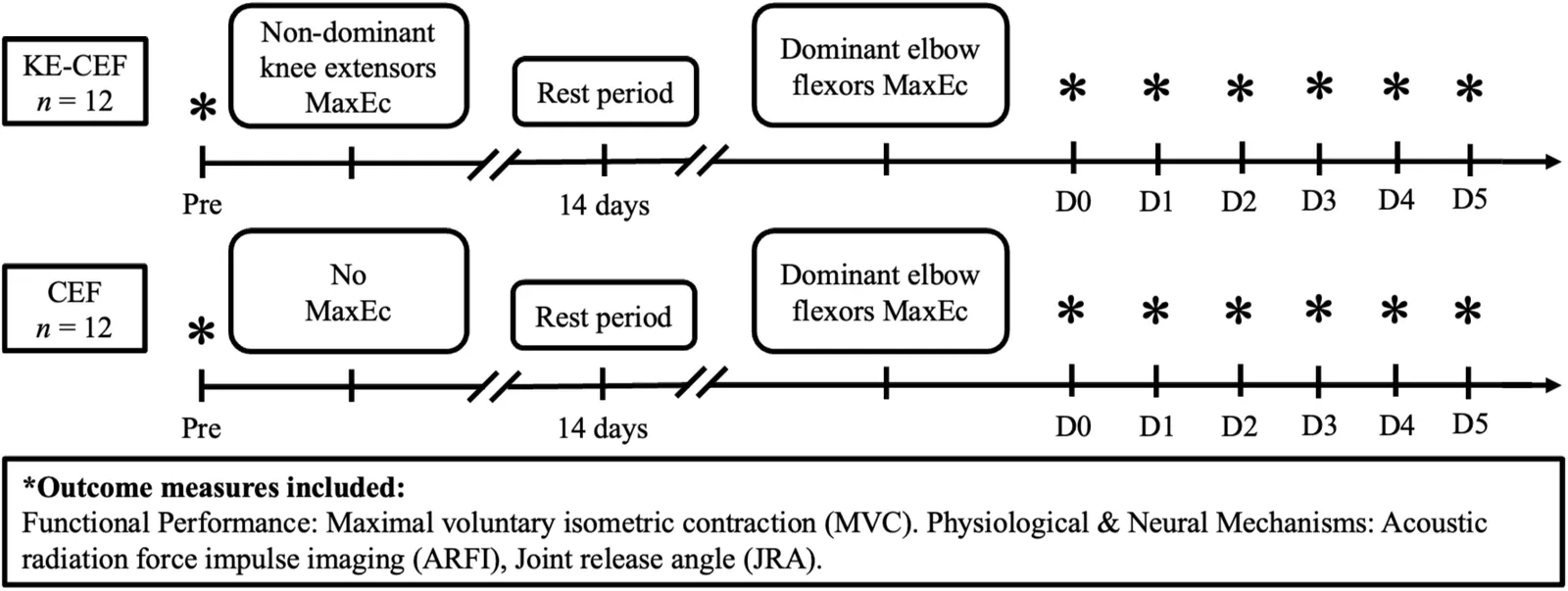
Study design flow chart. KE-CEF = Non-dominant knee extensors eccentric exercise followed by dominant elbow flexors eccentric exercise; CEF = Control followed by dominant elbow flexors eccentric exercise; MaxEc = Maximal eccentric exercise; * = Time points for outcome measures.

An *a priori* power analysis was conducted using G*Power 3.1.9.7 to determine the required sample size. Based on a two-way mixed-design ANOVA with repeated measures (Group × Time), assuming a small-to-moderate effect size (f = 0.21), derived from previous cross-limb and heterologous eccentric exercise literatures (Chen et al., 2021, 2023), which consistently demonstrate that distant cross-limb transfers yield smaller effect magnitudes (f = 0.18–0.25) compared to localized ipsilateral protective adaptations (f > 0.40), an alpha level (α) of 0.05, and a statistical power (1-β) of 0.80, the estimated minimum sample size required was 24 participants. Accordingly, we recruited 24 participants (12 per group). A *post hoc* sensitivity analysis performed in G*Power 3.1.9.7 indicated that with a total sample size of 24 (divided into 2 groups across 7 repeated measurements), the study possessed a statistical power of 1-β = 0.80 to detect a minimum effect size of f = 0.198 at an alpha level of α = 0.05. This demonstrates that our sample size was sufficiently sensitive to capture the hypothesized small-to-moderate cross-limb effects.

This sample size matches the statistical requirement, ensuring sufficient power to detect heterologous contralateral adaptations.

### 2.2 Participants

Twenty-four sedentary females aged 18–30 years (sedentary behavior defined as sitting for 4–8 h/day) participated in this study. All participants confirmed no involvement in any structured resistance or lower/upper-body exercise training for at least 6 months prior to enrollment. All participants confirmed no involvement in any structured resistance or lower/upper-body exercise training for at least 6 months prior to enrollment. To ensure participants were truly sedentary rather than active individuals with high sitting time, physical activity levels were quantified using the International Physical Activity Questionnaire Short Form (IPAQ-SF). Participants met the standard sedentary classification, reporting an average physical activity level of 412.5 ± 95.8 MET-min/week (well below the 600 MET-min/week threshold for moderate activity) and a daily sitting time of 6.5 ± 1.2 h/day.

Exclusion criteria included any musculoskeletal or neurological injuries within the past three months. All participants were informed of the study’s purpose, procedures, potential risks, and benefits, and provided written informed consent prior to participation. This study was conducted in accordance with the Declaration of Helsinki and was approved by the Research Ethics Committee of National Taiwan University (Protocol No. 202312EM073; approval date: 23 July 2024). All participant recruitment, screening, and experimental testing sessions were commenced in October 2024 and fully completed in June 2025, entirely within the active validity period of the ethical approval. During the experimental period, participants were instructed to refrain from vigorous exercise, resistance training, vitamin or high-protein supplementation, and any form of physical therapy to minimize confounding factors.

### 2.3 Procedures

#### 2.3.1 Familiarization and baseline

One week prior to the main experiment, all participants visited the laboratory for a familiarization session to minimize potential learning effects. During this visit, anthropometric data were collected, and participants were familiarized with the experimental equipment (Biodex System 4 Dynamometer) and testing procedures, including maximal voluntary isometric contraction (MVC), and joint angle reproduction tasks. Participants practiced submaximal eccentric contractions (approximately 50% of perceived maximal effort) to understand the movement trajectory but avoided maximal efforts to prevent pre-experimental muscle damage.

#### 2.3.2 Experimental protocol

The study was conducted over two main phases separated by a 14-day interval. In the first phase (Induction Bout), participants in the KE-CEF group performed the maximal eccentric knee extensor exercise using their non-dominant leg, whereas the CEF group did not perform this session and maintained their normal daily activities. Fourteen days after the induction phase, both groups returned to the laboratory for the second phase (Criterion Bout). Following a standardized warm-up consisting of 5 min of low-intensity joint mobilization, baseline measurements (Pre) were obtained. Subsequently, both groups performed the maximal eccentric elbow flexor exercise using their dominant arm. Outcome measures were assessed immediately post-exercise (D0) and at 24 h intervals for 5 consecutive days (D1–D5).

#### 2.3.3 Eccentric exercise settings

Maximal eccentric exercise was performed using a Biodex System 4 Pro Dynamometer (Biodex Medical Systems, Shirley, NY, USA). For the lower limb induction task (KE-CEF group only), participants performed 60 repetitions (10 sets of 6 repetitions) of maximal eccentric knee extension. The range of motion (ROM) was set from 0° (full extension) to 90° of flexion at an angular velocity of 30°/s. For the upper limb criterion task (both groups), participants performed 30 repetitions (5 sets of 6 repetitions) of maximal eccentric elbow flexion with the dominant arm. The ROM was set from 90° of flexion to 0° (full extension) at an angular velocity of 30°/s. For both protocols, a rest interval of 15 s was provided between repetitions, and a 2 min rest was allowed between sets. Participants were verbally encouraged to exert maximal force during each contraction.

### 2.4 Outcome measures

Outcome measures were categorized into three primary domains: strength performance, muscle morphology, and neuromuscular control.

#### 2.4.1 Strength measurements

Strength performance was assessed using both static and dynamic protocols. To evaluate static muscle strength, MVC was measured using a Biodex System 4 Pro Dynamometer (Biodex Medical Systems, Shirley, NY, USA). Participants performed three maximal isometric contractions of the elbow flexors, each lasting 3 s, with the elbow joint fixed at 90° of flexion. The highest peak torque (N·m) recorded across the three trials was used for analysis.

#### 2.4.2 Muscle morphology

Muscle structural changes were examined using ARFI imaging. Muscle stiffness was quantified using an ACUSON S2000 ultrasound system (Siemens Healthcare, Erlangen, Germany) equipped with a 9L4 linear array transducer. The Shear Wave Velocity (SWV, m/s) was measured at the mid-belly of the biceps brachii muscle. ARFI SWV is a validated, non-invasive acoustic technique directly proportional to shear modulus and muscle tissue stiffness (Kawama et al., 2022). In our laboratory, pre-experimental intra-operator reliability for biceps brachii SWV measurements demonstrated an Intraclass Correlation Coefficient (ICC) of 0.91 (95% CI: 0.82–0.96), establishing excellent measurement reproducibility. This value served as a direct indicator of tissue stiffness and structural integrity, reflecting the extent of muscle damage and edema.

#### 2.4.3 Neuromuscular control

Proprioceptive acuity and neuromuscular fatigue were assessed using the JRA test. During this assessment, the dynamometer passively moved the participant’s limb to randomly assigned target angles (30°) at a velocity of 20°/s. Upon release, participants were instructed to actively arrest the limb movement as quickly as possible. The deviation from the target angle was recorded to evaluate the precision of neuromuscular control. The JRA test evaluates dynamic proprioceptive motor control and sensorimotor braking capacity following sudden joint perturbation (Methenitis et al., 2023). Test-retest reliability for JRA target deceleration at 30° demonstrated high reproducibility in our baseline trials (ICC = 0.88, 95% CI: 0.79–0.94).

### 2.5 Statistical analysis

Statistical analyses were performed using GraphPad Prism 8 (GraphPad Software, San Diego, CA, USA). Data are presented as mean ± standard deviation (SD). For all variables, a two-way mixed-design ANOVA with repeated measures (Group × Time) was conducted first to evaluate the interaction effect, followed by main effects. When a significant interaction was detected, follow-up one-way repeated-measures ANOVAs were performed within each group across time. *Post-hoc* pairwise comparisons were conducted using Fisher’s Least Significant Difference (LSD) test. The use of protected LSD was chosen *a priori* as a planned *post-hoc* procedure given the primary protective ANOVA interaction, maintaining necessary statistical sensitivity across continuous recovery time points (D0–D5) without incurring severe Type II errors associated with over-conservative adjustments. Effect sizes were reported as partial eta squared ( $\eta_{\text{p}}^{2}$) for ANOVA effects and Cohen’s d for pairwise comparisons. The level of statistical significance was set at α = 0.05.

## 3 Results

### 3.1 Baseline homogeneity

No significant differences were observed between the KE-CEF and CEF groups at baseline for any outcome measures, including MVC, ARFI (Shear Wave Velocity, SWV), and JRA (*p* > 0.05). This indicates that the groups were homogeneous prior to the intervention (**Table 1**).

**Table 1:** Baseline homogeneity test for physical characteristics and all outcome measures.

Independent variables	KE-CEF (n = 12)	CEF (n = 12)	*P*-value
Strength measurement			
MVC Peak torque of elbow flexors (Nm/kg)	26.63 ± 5.56	31.54 ± 7.04	.168
Joint and muscle morphology assessments			
Muscle Stiffness (ARFI, m/s)	0.95 ± 0.13	0.96 ± 0.03	.085
Neuromuscular control			
JRA at 30° (°)	26.64 ± 1.42	26.81 ± 1.21	.380

Data are presented as mean ± standard deviation (SD). KE-CEF, Knee Extensor-Contralateral Elbow Flexor group; CEF, Control Elbow Flexor group; MVC, maximal voluntary isometric contraction; ARFI, acoustic radiation force impulse (measured by shear wave velocity); JRA, joint release angle. The significance level was set at α = 0.05.

### 3.2 Strength recovery (MVC)

Regarding MVC, a significant Group × Time interaction effect was observed (F_6, 132_ = 3.42, *p* = 0.004, $\eta_{\text{p}}^{2}$ = 0.135; *Post-hoc* at D0: d = 0.82). Significant main effects were also detected for Group (F_1, 22_ = 5.12, *p* = 0.034, $\eta_{\text{p}}^{2}$ = 0.189) and Time (F_6, 132_ = 28.45, *p* < 0.001, $\eta_{\text{p}}^{2}$ = 0.564). Follow-up one-way repeated-measures ANOVAs revealed significant time-course strength declines within both the CEF group (F_6, 66_ = 21.14, *p* < 0.001, $\eta_{\text{p}}^{2}=\;$0.658) and the KE-CEF group (F_6, 66_ = 9.87, *p* < 0.001, $\eta_{\text{p}}^{2}=\;$0.473). Subsequent LSD *post-hoc* tests demonstrated that immediately post-exercise (D0), the CEF group demonstrated a significantly greater decline in strength (57.67 ± 8.28%, approximately 42% loss) compared to the KE-CEF group (70.00 ± 19.85%, approximately 30% loss; *p* = 0.029, d = 0.82). A formal outlier evaluation using Grubbs’ test (α = 0.05) on the D0 MVC data within the KE-CEF group confirmed that no data points qualified as statistical outliers (p > 0.05), indicating that the higher variance reflects a broad physiological spectrum of individual responsiveness to cross-limb pre-conditioning. Furthermore, the KE-CEF group exhibited accelerated recovery, returning to near-baseline levels significantly earlier than the CEF group, which remained impaired until 96 h post-exercise (D4) (CEF: 80.67 ± 11.89% vs. KE-CEF: 89.17 ± 12.64%, *p* = 0.041, d = 0.69) (**Figure 2**).

**Figure 2:**
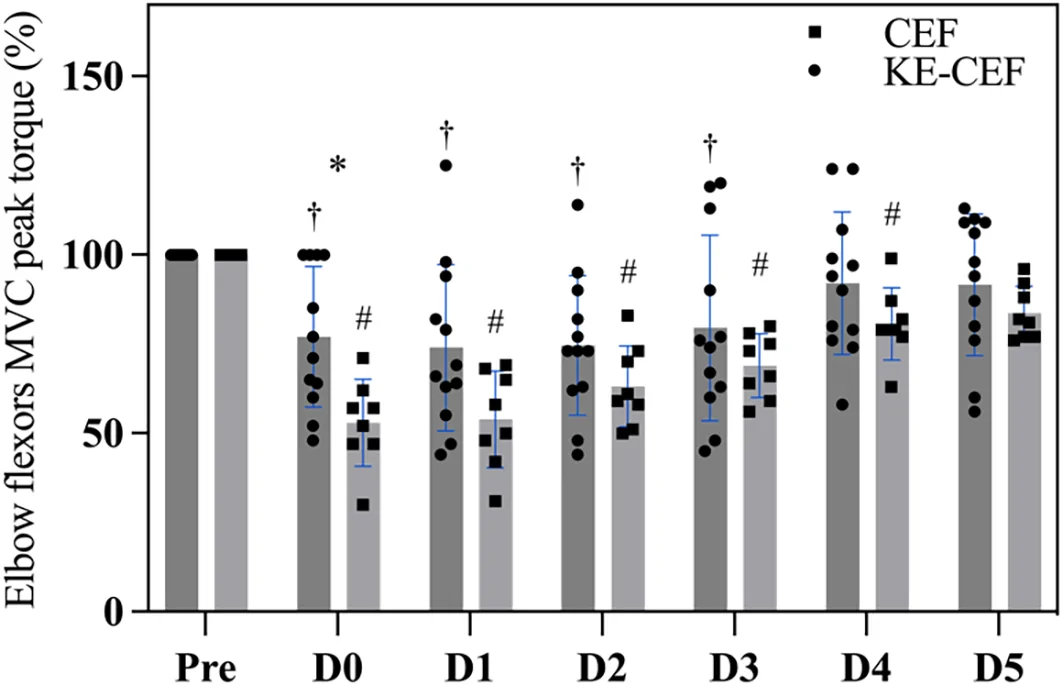
Changes in elbow flexors maximal voluntary isometric contraction torque. † indicates a significant difference from baseline within the KE-CEF group (*p* < 0.05); # indicates a significant difference from baseline within the CEF group (*p* < 0.05); * indicates a significant difference between the KE-CEF and CEF groups at that specific time point (*p* < 0.05).

### 3.3 Muscle stiffness (ARFI)

Changes in muscle stiffness (ARFI shear wave velocity) revealed a significant Group × Time interaction effect (F_6, 132_ = 5.18, *p* < 0.001, $\eta_{\text{p}}^{2}$ = 0.191, *Post-hoc* at D2 peak stiffness: d = 1.25). Main effects for Group (F_1, 22_ = 18.64, *p* < 0.001, $\eta_{\text{p}}^{2}$ = 0.459) and Time (F_6, 132_ = 34.12, *p* < 0.001, $\eta_{\text{p}}^{2}$ = 0.608) were also significant. Follow-up one-way ANOVAs confirmed significant time-course stiffness increases within both the CEF (F_6, 66_ = 28.91, *p* < 0.001, $\eta_{\text{p}}^{2}=0.724$) and KE-CEF groups (F_6, 66_ = 10.43, *p* < 0.001, $\eta_{\text{p}}^{2}=0.487$). LSD *post-hoc* comparisons showed that both groups exhibited a significant increase in stiffness from D0 to D4 compared to baseline (*p* < 0.05). However, between-group comparisons demonstrated that the CEF group showed significantly higher ARFI values than the KE-CEF group across D0 to D4 (*p* < 0.05, d = 0.65–1.25). At peak stiffness (D2), the CEF group reached 125.63 ± 6.99%, significantly higher than 108.25 ± 4.73% in the KE-CEF group (*p* < 0.001, d = 1.25).

Notably, a significant between-group difference was observed throughout the post-exercise period from D0 to D4 (*p* < 0.05). The CEF group demonstrated a significantly greater magnitude of increase in ARFI values compared to the KE-CEF group. For instance, at the peak of muscle stiffness (D2), the CEF group reached 125.63 ± 6.99%, which was significantly higher than the 108.25 ± 4.73% observed in the KE-CEF group. These results indicate that while both groups experienced structural damage and edema, the prior lower limb conditioning in the KE-CEF group significantly attenuated the severity of muscle stiffness in the heterologous upper limb (**Figure 3**).

**Figure 3:**
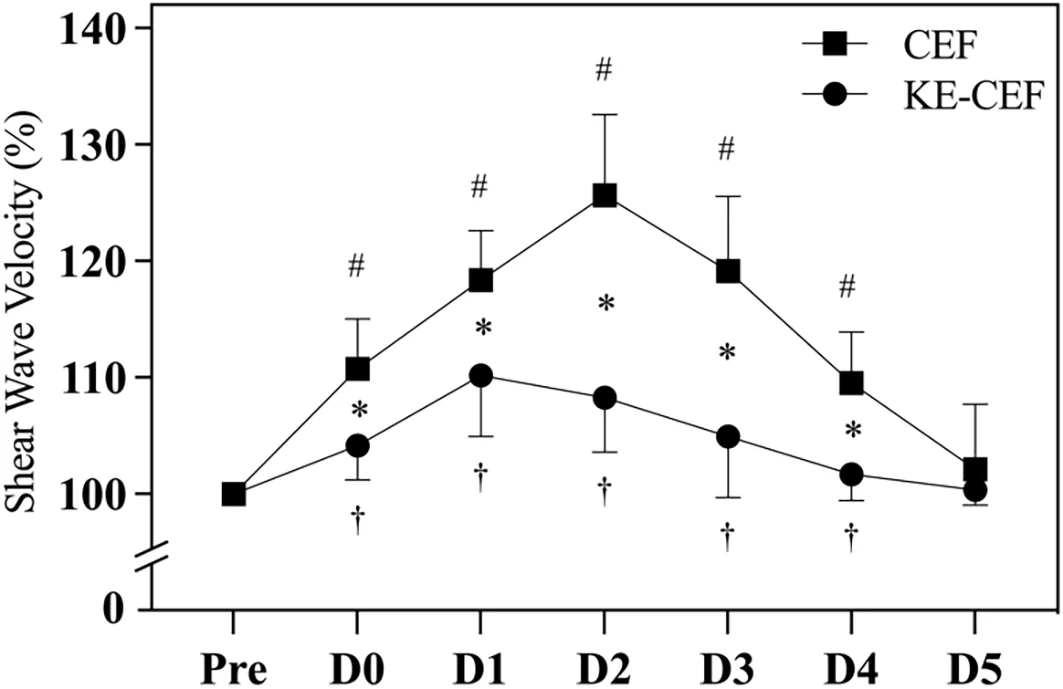
Changes in elbow flexors muscle stiffness (Shear Wave Velocity). † indicates a significant difference from baseline within the KE-CEF group (*p* < 0.05); # indicates a significant difference from baseline within the CEF group (*p* < 0.05); * indicates a significant difference between the KE-CEF and CEF groups at that specific time point (*p* < 0.05).

### 3.4 Neuromuscular control (JRA)

Neuromuscular control and proprioceptive acuity were evaluated using the JRA test demonstrated a significant Group × Time interaction effect (F_6, 132_ = 3.89, *p* = 0.001, $\eta_{\text{p}}^{2}$ = 0.150, *Post-hoc* at D2: d = 1.48), alongside significant main effects for Group (F_1, 22_ = 8.76, *p* = 0.007, $\eta_{\text{p}}^{2}$ = 0.285) and Time (F_6, 132_ = 4.12, *p* = 0.001, $\eta_{\text{p}}^{2}$ = 0.158) (**Figure 4**). Follow-up one-way repeated-measures ANOVAs revealed a significant time effect within the CEF group (F_6, 66_ = 6.45, *p* < 0.001, $\eta_{\text{p}}^{2}=0.370$), but no significant time variation in the KE-CEF group (F_6, 66_ = 0.82, *p* = 0.558, $\eta_{\text{p}}^{2}=0.069$). *Post-hoc* LSD comparisons showed that the CEF group showed significant deviations from baseline from D1 to D3 (*p* < 0.01), reaching 24.75 ± 1.55° at D2 (*p* = 0.003). In contrast, the KE-CEF group maintained baseline accuracy throughout D0–D5 (26.58 ± 0.78° at D2 vs. baseline 26.64 ± 1.42°, *p* > 0.05). Between-group *post-hoc* analysis confirmed a significant difference at D2 (*p* = 0.001, d = 1.48).

When comparing the two groups, a significant between-group difference was observed at D2 (*p* = 0.001). These results indicate that the prior lower limb conditioning effectively preserved heterologous neuromuscular control and mitigated proprioceptive impairments in the upper limb during the peak fatigue phase.

**Figure 4:**
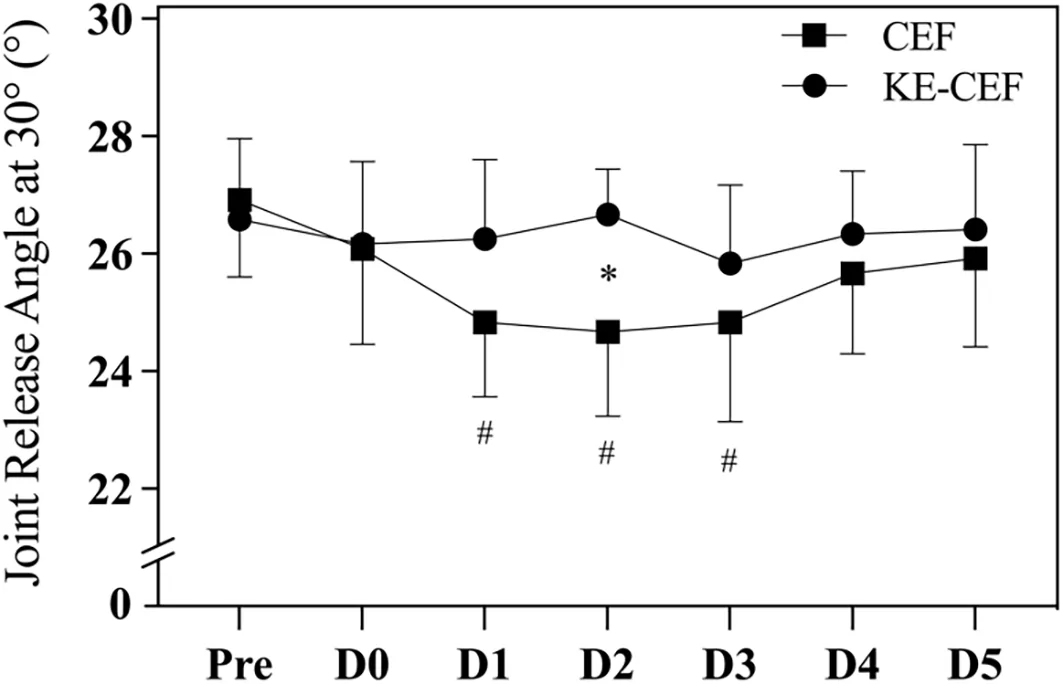
Changes in elbow flexors joint release angle at 30°. # indicates a significant difference from baseline within the CEF group (*p* < 0.05); * indicates a significant difference between the KE-CEF and CEF groups at that specific time point (*p* < 0.05).

## 4 Discussion

The primary objective of this study was to investigate whether a high-intensity bout of eccentric knee extensor exercise could induce a heterologous contralateral repeated bout effect (CL-RBE) to attenuate EIMD and fatigue in the elbow flexors among sedentary young women. The results strongly supported our hypothesis. We demonstrated that prior lower limb conditioning significantly mitigated the loss of MVC strength, attenuated exercise-induced muscle stiffness (ARFI), and entirely preserved neuromuscular control (JRA) in the heterologous upper limb. These findings provide compelling novel evidence for the existence of heterologous CL-RBE, challenging the current consensus and offering new insights into inter-limb neural and systemic adaptations (Pincheira et al., 2018).

### 4.1 Attenuation of strength loss and accelerated recovery

The protective effect of the lower limb induction bout was clearly evident in the preservation of static strength. Following the criterion bout, the CEF group experienced a substantial MVC deficit (approximately 42% loss), whereas the KE-CEF group demonstrated a significantly blunted decline (approximately 30% loss) at D0 (*p* = 0.029). Because the initial mechanical deficit was significantly attenuated, the KE-CEF group reached near-baseline levels earlier (by D4), although both groups subsequently followed a generally comparable recovery trajectory across D1–D5. This immediate preservation of strength at D0 strongly suggests that the lower-limb pre-conditioning acted to mitigate initial sarcomere strain and microstructural disruption during the eccentric task itself, likely via neural motor overflow or enhanced motor unit synchronization, rather than solely accelerating post-damage cellular repair. Interestingly, our findings contradict several recent investigations. For instance, Brown et al. (2023), Chen et al. (2021), and Jeon et al. (2022) reported that maximal eccentric exercise of one limb failed to confer protection to a heterologous muscle group (e.g., leg to arm, or arm to leg). The discrepancy between our results and these prior studies may be attributed to methodological and demographic differences. Most notably, previous studies predominantly recruited male participants. Females and males exhibit distinct neuromuscular control strategies, motor unit firing rates, and hormonal profiles (Romero-Parra et al., 2021). It is plausible that female neuromuscular and systemic systems are more susceptible or responsive to the global “motor overflow” and pre-conditioning effects induced by intense exercise of a large muscle mass (such as the knee extensors). Furthermore, the high volume of our induction stimulus (60 maximal eccentric contractions) may have been necessary to surpass the threshold required to trigger a systemic heterologous response. The decision to utilize unequal contraction volumes (60 repetitions for knee extensors vs. 30 repetitions for elbow flexors) was based on the distinct muscle mass and structural tolerance differences between the upper and lower limbs in sedentary cohorts. Furthermore, the 15-s inter-repetition rest interval successfully isolated mechanical stress from confounding metabolic accumulation. The robust EIMD symptoms observed in the control group (e.g., ~42% MVC loss and prolonged ARFI stiffness) confirm that the upper limb criterion bout provided a highly sufficient damage stimulus, eliminating the possibility of an artificially amplified protective appearance.

### 4.2 Modulation of muscle stiffness and systemic adaptations

ARFI imaging provided critical insights into the structural integrity of the biceps brachii. Increased shear wave velocity is a direct indicator of muscle stiffness, reflecting edema, inflammatory infiltration, and structural disruption of sarcomeres typical of EIMD (Kawama et al., 2022). The CEF group exhibited a pronounced increase in stiffness, peaking at 125.63% at D2. In stark contrast, the KE-CEF group demonstrated only a minimal, transient elevation (peaking at 108.25%). This remarkable attenuation of muscle stiffness is consistent with the hypothesis that systemic adaptive mechanisms were involved, thereby creating a global pre-conditioned environment that successfully mitigated subsequent strain in the remote upper-limb muscles. Importantly, the immediate attenuation of MVC loss at D0 suggests that initial mechanical sarcomere disruption was mitigated, likely mediated by altered neuromuscular recruitment strategies or pre-conditioned muscle strain distribution. Concurrently, the pronounced suppression of post-exercise muscle stiffness (ARFI) across D1–D4—particularly at the 48-h peak (D2)—indicates that systemic pre-conditioning played an essential secondary role in blunting downstream inflammatory cascades and interstitial edema, thereby attenuating the severity of delayed muscle stiffness rather than solely governing contractile recovery kinetics. EIMD triggers an acute inflammatory cascade. We hypothesize that the severe unaccustomed eccentric exercise of the large lower limb muscles 14 days prior may have acted as an “immunological vaccine” for the skeletal muscle system. This pre-conditioning likely upregulated circulating systemic factors, such as cellular memory markers or anti-inflammatory cytokines, creating a blunted inflammatory environment globally (Calvo-Rubio et al., 2024; Sulistyarto et al., 2022). Consequently, when the elbow flexors were subsequently subjected to EIMD, the secondary damage phase and edema were significantly suppressed. However, the precise structural signaling pathways require further elucidation. However, the precise structural signaling pathways require further elucidation. It is plausible that the 60 MaxEc induction protocol induced sufficient localized mechanical strain and neuromuscular fatigue in the large knee extensor mass to act as a potent systemic stimulus, thereby upregulating circulating factors or neural drive and creating a global pre-conditioned environment that attenuated subsequent exercise-induced stiffness in the remote upper-limb muscles.

### 4.3 Preservation of neuromuscular control and neural adaptations

Perhaps the most striking finding of this study is the complete preservation of neuromuscular control in the KE-CEF group. EIMD is well-documented to impair proprioceptive acuity due to the disruption of mechanoreceptors (such as muscle spindles) and altered central processing of afferent feedback (Methenitis et al., 2023). As expected, the CEF group showed significant proprioceptive deficits, with the JRA at 30° deviating significantly from baseline during the peak fatigue phase (D1–D3). This exercise-induced JRA decrease represents a distinct directional overshoot past the target angle, which reflects a compromised ability to rapidly process proprioceptive feedback and execute immediate braking commands. Such deficits are heavily consistent with muscle spindle microstructural disruption and transient impairments in central sensorimotor integration or efference copy evaluation following severe eccentric strain. Conversely, the KE-CEF group maintained remarkable stability, exhibiting no significant deviations across all post-exercise time points. This absolute preservation of joint position sense may suggest the potential involvement of central neural pathways for neural mechanisms underlying heterologous CL-RBE. The intense central drive required to perform 60 maximal eccentric contractions of the knee extensors may have induced global neuroplasticity. According to the “cross-education” and “motor overflow” theories, unilateral heavy resistance training can enhance cortico-spinal excitability and optimize motor unit recruitment strategies beyond the exercised limb (Mirto et al., 2025; Valdés et al., 2025). While the functional data suggest that lower-limb eccentric exercise may fortify the neural pathways responsible for processing proprioceptive feedback and executing motor commands, this interpretation warrants direct neurophysiological investigation to confirm precisely how central pathways modify sensorimotor signaling and shield the upper limb from exercise-induced impairments.

### 4.4 Limitations

While this study provides novel evidence for heterologous CL-RBE, several limitations must be acknowledged. First, the sample was restricted to sedentary young females, meaning the results may not be generalizable to males, older adults, or highly trained athletes. Second, while a comprehensive familiarization session was conducted for all participants to minimize learning effects, the current study lacked a dynamic ‘sham exercise’ or ‘placebo’ control group during the 14-day induction period. Third, while ARFI and JRA provide excellent mechanical and functional measures of tissue stiffness and neuromuscular fatigue, we did not collect direct circulating biomarkers (e.g., blood creatine kinase, inflammatory cytokines) or advanced neurophysiological data (e.g., electromyography, transcranial magnetic stimulation) to definitively isolate the underlying systemic and central pathways. Future research should incorporate low-intensity concentric or isometric sham protocols to rule out potential psychological expectancy effects, as well as include these blood-borne and electrophysiological measurements to further confirm the precise transfer mechanisms.

## 5 Conclusion

This study provides novel and compelling evidence that a single bout of high-intensity eccentric exercise of the lower limbs induces a robust heterologous contralateral protective effect on the upper limbs in sedentary young women. Specifically, this pre-conditioning effectively attenuated the loss of muscle strength, mitigated exercise-induced muscle stiffness and structural damage, and entirely preserved proprioceptive neuromuscular control during the peak fatigue phase. These findings challenge the traditional view that the repeated bout effect is strictly localized or homologous, highlighting the significant role of systemic and global neural adaptations—such as inflammatory modulation and “motor overflow”—in cross-limb protection. From a clinical and athletic perspective, understanding this global cross-limb interaction opens innovative avenues for rehabilitation. For individuals experiencing upper limb injuries, unilateral immobilization, or severe fatigue, incorporating high-intensity lower body resistance training may serve as a viable pre-conditioning strategy to preserve neuromuscular integrity and accelerate functional recovery in distant muscle groups.

## Data Availability

The original contributions presented in the study are included in the article/supplementary material. Further inquiries can be directed to the corresponding author.
